# A Modern Lettre De Cachet

**Published:** 1902-05-24

**Authors:** 


### The Hospital

A Journal of

The Medical Sciences and Hospital Administration.

Vol. XXXII.—No. 817. Edited by Sir Henry Burdett, K.C.B., and Solomon C. Smith, M.D., M.R.C.P. [May 24, 1902.

### A Modern "Lettre de Cachet."

The question how far we may properly go in the compulsory removal of infectious cases is becoming one of considerable importance. In the eyes of the sanitary authorities compulsory removal is the very key-note of progress, but from the rights-of-the-subject point of view a very different aspect of the question is presented, and we confess that we are sometimes inclined to doubt whether the law would long remain as it is were its bearings and possibilities thoroughly appreciated by the public at large. Under Section 124 of the Public Health Act, any person who is suffering from "any dangerous infectious disorder and is without proper lodging and accommodation," may, on a certificate signed by a legally qualified practitioner, be removed to a hospital if such a hospital exists within a "convenient distance." It was long held that the above word "proper" referred only to the patient's own well-being, but during recent years it has been held to include the question of infection to others. Thus it may happen that a medical man in certifying may set up his own, perhaps extravagant, definition as to what is to be considered "proper lodging and accommodation," and on the strength of his certificate, without any further evidence (except as to the existence of a hospital at which he can be received), and without any notice of the application being sent to the responsible relatives an order may be obtained from a Justice, and the patient may be compulsorily removed to hospital. It may then, as a climax, turn out that the patient has never had the disease at all, and yet, as has lately been shown in the courts, there is no redress. We need hardly say that this condition of the law is a very serious matter. If the sanitary law were always administered by people of common sense; if there were no "scares"; if the hospitals, which the law calls "suitable," were always good and well managed; if what the law calls a "convenient" distance were interpreted to mean such a distance as the patient could be removed *without suffering injury*; and if the diagnosis of the disease were so straightforward a matter that there was a reasonable probability of the medical man's certificate being correct, the public would not have so much cause for anxiety.

But it is notorious that there is no security whatever in regard to any one of these particulars, and that there is no folly which may not be committed in the name of sanitation during times of panic. As to the lack of common sense sometimes displayed by sanitary authorities we need say nothing. As to the hospitals some are good; on the other hand, some are wretchedly bad. The majority of them are entirely without resident medical officers, the nurses in many are untrained, the equipment is just such as to escape open scandal, and the medical attendance has to be given by the medical officer of health in such time as he can spare amid the extra work thrown upon him by an epidemic.

As to what the law calls a "convenient distance" we are afraid that in the interpretation of this term by sanitary officials "the wish is" often "father to the thought." The distance which patients are being carried in ambulances is a growing danger, and it is a fact which should not be forgotten that as the law is now being administered serious syncope during removal by ambulance is by no means unknown, and that, in the words of a well known medical officer, "it is not at all uncommon to find diphtheria patients exhausted and even collapsed on admission." This may be unavoidable, nevertheless the danger run by the patient must enter into the consideration of the case when it is a matter of his being removed purely on public grounds without any compensating advantage to himself. Lastly, as to the diagnosis, take the example of small-pox. We do not know the exact figures for the present outbreak in London, but it is notorious that the errors of diagnosis have been so frequent that the various metropolitan boroughs have had to appoint special medical diagnosticians with the object of minimising, as far as possible, the scandal created by the removal of patients who were not suffering from small-pox. If, however, we go back to the outbreak in 1894, we find that out of 1,263 cases removed to the wharves under medical certificate as cases of small-pox 155, or 12·2 per cent., were not suffering from that disease. Still worse were the figures in 1897, a time when London was comparatively free from the disease, for so great had this evil of mal-diagnosis then become that out of 36 cases certified as small-pox during the year, only six had the disease. Under these circumstances how far is it right that compulsory and immediate removal should be enforced by sanitary officials without notice, without a chance of obtaining legal advice or bringing forward rebutting evidence, or of showing that the "accommodation" is or can be rendered "proper," without a chance even of obtaining a second opinion on the medical aspects of the case? As a profession we naturally feel grateful for the touching confidence reposed in us by the public. Whether it is wise to repose such faith in the single unassisted judgment of any official is another matter.

